# Non-contrast cardiac MRI for tissue characterization in patients with end stage renal disease

**DOI:** 10.1186/1532-429X-18-S1-P241

**Published:** 2016-01-27

**Authors:** Tori A Stromp, Joshua C Kaine, Kristin N Andres, Linyuan Jing, Brandon K Fornwalt, Steve W Leung, Vincent L Sorrell, Moriel Vandsburger

**Affiliations:** 1 Physiology, University of Kentucky, Lexington, KY USA; 2 Saha Cardiovascular Research Center, University of Kentucky, Lexington, KY USA; 3 College of Medicine, University of Kentucky, Lexington, KY USA; 4 Institute for Advanced Application, Geisinger Health System, Danville, PA USA; 5 Gill Heart Institute, University of Kentucky, Lexington, KY USA

## Background

**Figure 1:**
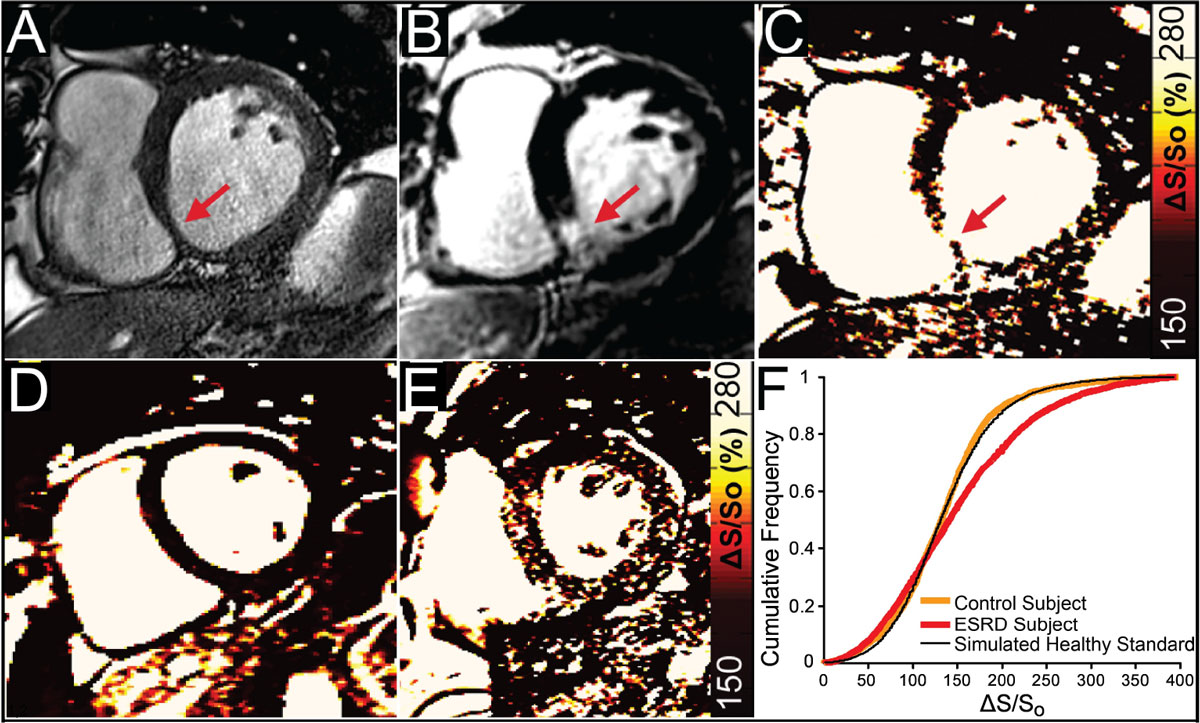
**Tissue characterization with MT-weighted bSSFP**. (A) End diastolic anatomical reference and (B) late gadolinium enhancement (LGE) image from a patient with chronic myocardial infarction reveal an area of fibrotic scar tissue (red arrow). (C) The corresponding map of ΔS/S_o_ acquired using MT-weighted CMR demonstrates elevated ΔS/S_o_ in the area identified as enhanced at LGE (images reprinted from prior validation study [1,2]). (D) Map of ΔS/S_o_ for a healthy control revealts low values throughout the left ventrical. (E) In contrast, a maps of ΔS/S_o_ in an ESRD patient reveals diffusely elevated values throughout the left ventricle, with several focal areas demonstrating values consistent with tissues that would enhance at LGE. (F) Representative cumulative distribution frequency plots of ΔS/S_o_ values for participants in D (orange) and E (red). For each participant a simulated healthy standard distribution (black) was used to adjust for differences in heart size. A one-sided Komogorov-Smirnov test revealed a significant (p < 0.001) rightward shift in the ESRD patient (negative differential statistic = 14%) compared to no observable difference in the control subject (2%, N.S. vs healthy standard).

End stage renal disease (ESRD) patients suffer high cardiovascular mortality rates with fibrosis-induced arrhythmia recognized as a leading contributor. Since ESRD patients are contraindicated to standard gadolinium-based fibrosis imaging, non-contrast cardiac magnetic resonance imaging (CMR) techniques could improve diagnosis and empower evaluation of emerging anti-fibrotic therapies. With remodeling and increased extracellular water volume, a corresponding and measurable loss of magnetization transfer (MT) occurs. We previously demonstrated the ability to identify tissue that enhances with gadolinium based on differences between pairs of differentially MT-weighted balanced steady state free precession (bSSFP) images^1,2^ (Figure 1A-C). In this study, we seek to apply non-contrast MT-based tissue characterization in ESRD patients.

## Methods

ESRD patients on routine hemodialysis and healthy controls (n = 9/group to date) were imaged on a 1.5T Siemens Aera scanner. Pairs of prospectively gated cine bSSFP images were obtained at flip angles of 50 and 45° from base to apex [TR/TE=35.64/1.36 ms, FOV=260 × 260 mm^2^, Matrix=256 × 256, Thickness=8 mm, phases set to fill the cardiac interval]. Maps of ΔS/S_o_ were generated as ΔS/S_o_=(S_45_-S_5_)/S_5_*100 (%), where S_i_ is the signal intensity per voxel at flip angle *i*. We analyzed cardiac structure and global function. A custom feature tracking algorithm measured circumferential and longitudinal strains. The distribution of ΔS/S_o_ values across all controls was used to define a reference standard. To account for variations in heart size, this distribution was dynamically resized to simulate a cumulative distribution function of ΔS/S_o_ matching the number of voxels per individual heart. Each subject's observed ΔS/S_o_ distribution was compared to the appropriately-sized healthy standard using a one-sided Kolmogorov-Smirnov (KS) test.

## Results

While septal wall thickness was heightened in ESRD patients (Table 1), ejection fraction and peak circumferential and longitudinal strains were similar to controls. A sample ΔS/S_o_ map in an ESRD patient (Figure 1E) demonstrates scattered enhancement compared to a healthy control (Figure 1D). Corresponding ΔS/S_o_ distributions (Figure 1F) reveal a right skew in the ESRD patient, consistent with tissue remodeling. KS analysis revealed a trend toward greater rightward shift for ESRD patients (avg=13%, range: 3-48%) than controls (avg=4%: 0-12%, p = 0.12 vs. ESRD) when compared to the healthy standard. Four ESRD patients and only 1 control displayed ΔS/S_o_ distributions that diverged from the standard distribution by more than 10%.

**Table 1:** Participant Characteristics, Function, and Mechanics Results

Variable	Control (n = 9)	ESRD (n = 9)
Age (yrs.)	52.0 ± 5.0	53.3 ± 17.0
Male	6	5
Dialysis Vintage (yrs.)	n/a	5.5 ± 2.3
BMI (kg/m^2^)	23.8 ± 1.5	31.3 ± 5.3**
Heart Rate at MRI (bpm)	58 ± 13	71 ± 10*
Ejection Fraction (%)	56.4 ± 4.7	62.6 ± 7.7
End Diastolic Septal Thickness (mm)	8.8 ± 0.1	13.2 ± .3**
Peak Circumferential Strain	29.6 ± 6.5	30.2 ± 6.6
Peak Longitudinal Strain	22.1 ± 3.4	20.9 ± 3.5
Mean per Subject ΔS/S_o_ (%)	135.9 ± 12.8	150.6 ± 25.1

BMI: Body Mass Index, *p < 0.05, **p < 0.01 vs. ESRD.

## Conclusions

MT-weighted bSSFP imaging revealed a promising trend towards elevated ΔS/S_o_ values in ESRD patients compared to controls, despite preserved contractile function. Analysis of ΔS/S_o_ distributions may provide a rapid and endogenous mechanism for myocardial tissue characterization in ESRD patients. Ongoing participant recruitment will increase sample sizes and may reveal additional pathologies in ESRD patients.

## References

[1] Stromp T (2014). Circulation.

[2] Stromp T, et al: JCMR Under review following revision.

